# Alteration of the Condylar Oral Bone in Obese and Gastric Bypass Mice

**DOI:** 10.1007/s00223-020-00732-0

**Published:** 2020-08-01

**Authors:** Nicolas Colsoul, Carlos Marin, Katrien Corbeels, Greet Kerckhofs, Bart Van der Schueren, Katleen Vandamme

**Affiliations:** 1 Biomaterials – BIOMAT, Department of Oral Health Sciences, KU Leuven, Leuven, Belgium; 2 Prometheus – Division of Skeletal Tissue Engineering Leuven, KU Leuven, Leuven, Belgium; 3 Department of Development and Regeneration, Skeletal Biology and Engineering Research Center, KU Leuven, Leuven, Belgium; 4 Department of Chronic Diseases, Metabolism and Ageing (CHROMETA), Clinical and Experimental Endocrinology, KU Leuven, Leuven, Belgium; 5 Department of Material Science and Engineering, KU Leuven, Leuven, Belgium; 6 Biomechanics Lab, Institute of Mechanics, Materials, and Civil Engineering, UCLouvain, Louvain-la-Neuve, Belgium; 7 Institute for Experimental and Clinical Research, UClouvain, Woluwe, Belgium

**Keywords:** Type 2 diabetes, Roux-en-Y gastric bypass, Mandibular condyle, Bone composition, Cancellous bone, Raman spectroscopy

## Abstract

Obesity is the main cause of type 2 diabetes mellitus (T2DM). Roux-en-Y gastric bypass (RYGB) surgery is an effective treatment for this obesity-related health problem. However, the adverse effects of T2DM on bone tissue persist or even aggravate after this surgical procedure. As studies on the mandibular condyle bone are scarce, the aim of the present study was to assess its compositional characteristics in T2DM and RYGB conditions. Thirty-two male C57BL/6 mice at 8 weeks of age were randomly assigned to receive either a high-fat or low-fat diet. After 14 weeks of high-fat diet intake, seven obese mice were subjected to RYGB surgery. All animals were euthanized at the age of 30 weeks. Mandibular bones were removed and the trabecular condyle region was assessed using Raman spectroscopy. A decreased mineralization was observed for both T2DM and RYGB condyle bones when compared to controls, with elevated carbonate substitutions for the RYGB group. No compositional differences in crystallinity and presence of advanced glycation end products were found between the groups, with the exception of an increased presence of N-carboxymethyl-lysine in RYGB bone compared to their T2DM counterpart. Site-specific measurements revealed a non-uniform bone composition, with increasing mineralization and carbonate substitutions towards the centre of the mandibular condyle. T2DM and RYGB surgery affect the mandibular condyle bone quality, as investigated at compositional level. Assessment of bone structural properties and remodelling should be carried out to further explore the effects of T2DM and RYGB surgery on this skeleton area.

**Electronic supplementary material:**

The online version of this article (10.1007/s00223-020-00732-0) contains supplementary material, which is available to authorized users.

## Introduction

Diabetes is a defiant health problem in the twenty-first century, globally involving 451 million people in 2017 [6]. The majority is affected by type 2 diabetes mellitus (T2DM) [38], an endocrine disease characterised by hyperglycemia and beta cell dysfunction [37]. Chronic presence of hyperinsulinaemia along with glycaemic fluctuations result in excessive and accelerated formation of advanced glycation end products (AGEs) [45], and is known to adversely affect the musculoskeletal system [7]. Among many of the T2DM-induced complications, a higher bone fracture risk is evidenced and attributable to an impairment of structural, mechanical and compositional characteristics of the bone tissue [7].

The overall bone strength is to a large extent determined by the bone mineral density (BMD), and is often normal to high in T2DM patients [44]. Trabecular bone strength, however, may be more dependent on bone quality as this area is more susceptible to microdamage, turnover and mineralization alterations [2, 30]. The reduction of bone strength in T2DM is co-caused by a deteriorated bone quality induced by molecular changes through AGEs such as pentosidine and N-carboxymethyl-lysine (CML), which are able to form non-enzymatic cross-links between collagen molecules under the hyperglycemic conditions in T2DM [17, 34].

For the assessment of the chemical composition of bone, Raman spectroscopy is an appropriate technique that offers a superior resolution compared to Fourier transform infrared [19, 29]. By applying Raman spectroscopy on bone specimens, major spectral fingerprints that correspond to the mineral and organic components can be clearly separated [4, 9] and enable the assessment of collagen maturity, carbonate substitution, crystallinity and mineralization degree as measures of bone quality [16, 29]. A recent study of Marin et al. [24] used Raman spectroscopy to reveal that the femoral cortical area of obese and gastric bypass-treated mice is subject to AGE accumulation. Regarding mineralization, carbonate substitution and crystallinity, lean and diabetic obese groups presented similar outcomes in the cortical bone, indicating that the compositional characteristics of this area are not affected by T2DM [24]. In the trabecular bone, less carbonate substitution was seen in obese and bypass animals, aside from decreased mineralization and stiffness in the latter [24]. Hence, compositional bone alterations do occur in the diabetic state exerting a negative impact on the bone quality, which seem not to be reversed by a gastric bypass operation [24].

T2DM also affects the jaw bone [26]. In the mandibular condyle, trabecular bone can be found [18]. Onoyama et al. [31] showed that T2DM decreases trabecular BMD and induces morphological changes in terms of trabecular connectivity, causing a lower bone strength. Kim et al. [21] showed that the oral trabecular bone volume is not affected by type 1 diabetes, whereas a negative effect was seen in the tibia, indicating a different response relative to the embryonic origin (i.e. neuroectoderm for the mandible and mesoderm for the tibia).

In spite of these findings, research regarding T2DM and the jaw bone is extremely scarce. Therefore we examined the effect of T2DM on the mandibular condyle of diet-induced obese (DIO) C57BL/6 male mice, with emphasis on its composition using Raman spectroscopy. The influence of gastric bypass surgery on jaw bone alterations was also investigated, as bypass surgery is an effective treatment for obesity and T2DM [36]. As a secondary aim, we assessed whether differences between the Raman data could be attributed to a specific site within the condyle. Rationale of the current study was to discern if the known detrimental effects of diabetes on long bones also apply to the mandibular condyle bone, and if bariatric surgery also affects the oral condylar bone.

## Materials and Methods

### Animals and Diets

**Fig. 1:**
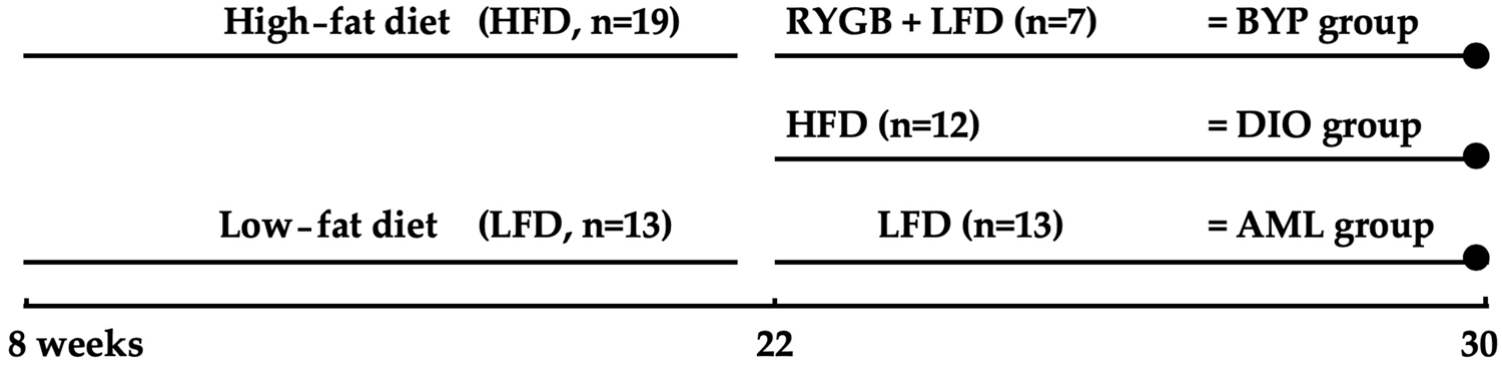
Classification of animals according to diet

All experimental procedures were approved by the Ethics Committee of the University of Leuven (P068/2016 and P101/2014). A total of 32 male mice, substrain C57BL/6 purchased at Janvier Labs (Le Genest-Saint-Isle, France), received a normal diet until the age of 8 weeks. Subsequently, mice were randomly divided into two diet groups, namely an age-matched lean (AML, *n* = 13) and a diet-induced obese (DIO), *n* = 19) group. Low-fat diet (5%fat, 0.9% Ca^2+^, 1500 International Units Vitamine D/kg, Research Diets, Inc.—New Brunswick, NJ, USA: D12450-B; open source) was administered to the AML mice for 22 weeks (Fig. 1). High-fat diet (Research Diets, Inc.: D12492) was administered to DIO mice for an initial period of 14 weeks, whereafter 7 DIO mice were randomly assigned to a new group (bypass group: BYP) and were subjected to Roux-en-Y gastric bypass surgery (RYGB). The RYGB surgical procedure is described in detail by Vangoitsenhoven [42]. Low-fat diet (E15051 EF R/M, Ssniff Gmbh, Soest, Germany) with a composition of 0.9% calcium and 1500 IU/kg vit D3 was provided for the 8 weeks post-surgery. The remaining 12 obese mice continued the high-fat diet intake. A similar animal and diet setup has been used in a study from [24, 25], confirming the diabetic state after 22 weeks of high-fat diet intake with significant increase in bodyweight and fasting blood glucose compared to lean controls.

### Bone Tissue Collection

Animals were euthanized at the age of 30 weeks by using carbon dioxide administration. Mice heads were collected and underwent a fixation treatment with paraformaldehyde 2% for 24 h. Thereafter, the samples were transferred to a phosphate-buffered saline solution and kept at 4 °C. Mandibular bones were separated at the midline and dissected. Hemi-mandibles were secured onto a support with the use of a light-curing dental bonding system (Clearfil SE Bond 2 Primer, Kuraray, New York, USA) and dental composite (G-aenial flo 30 μm PMMA 3 wt% A2, GC America, Illinois, USA). A cut through the longitudinal axis at the middle of the condyle was performed using a diamond blade of 102 × 0.3 × 12.7 mm (230CA), with the Accutom-50 device (Struers, Ballerup, Denmark). After cutting, the samples were wrapped in aluminium foil and stored at − 20 °C until Raman analysis.

### Raman Spectroscopy

**Fig. 2:**
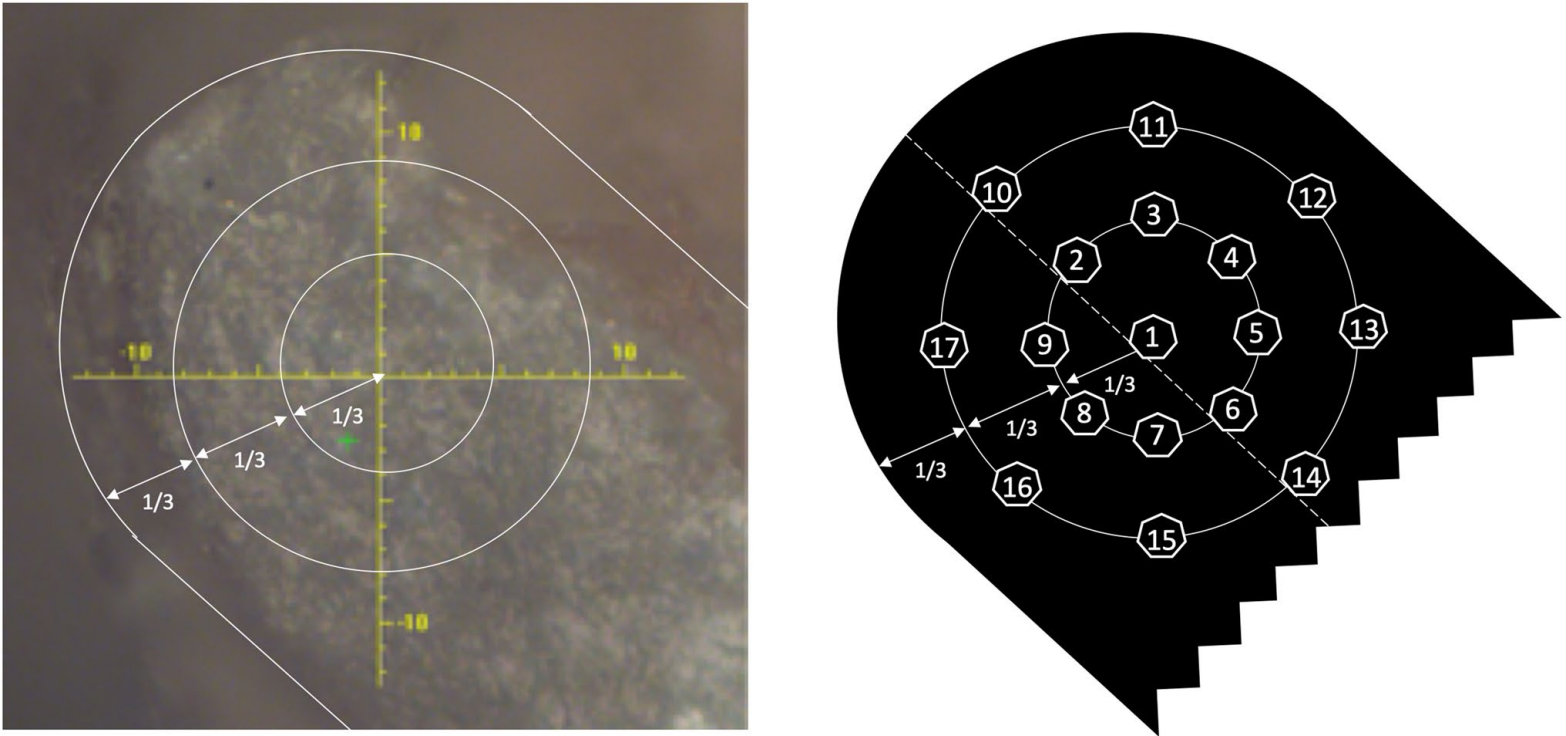
Measurement positions as determined using magnification ×20 (left) and illustrated in an anatomical drawing (right). The longitudinal axis of the sample is represented by the dotted line

Imaging of the samples was performed using a Raman micro-spectrometer (SENTERRA, Bruker, Massachusetts, USA). Raman outcomes of distinct bone types display peaks at similar positions but with different peak intensities [12]. To enable comparison of samples originating from different groups, measurement positions were standardised and covered the entire trabecular condyle area. A total of 17 measurements per sample was performed as follows (Fig. 2):

- *Measurement 1* in the centre of an imaginary circle located at the outer border of the condyle, beneath the cartilage;
- *Measurements 2 till 9* on a circle at radius 1/3 distance from the centre of this imaginary circle and with an eight stroke difference (clockwise) between the measurements, starting with measurement 2 located on the longitudinal axis of the condyle through the centre of the imaginary circle (displayed as a dotted line in Fig. 2), and closest to the outer edge of the condyle;
- *Measurements 10 till 17* idem on a circle at radius 2/3 distance.

Raman spectra were acquired with laser settings of *λ* = 785 nm, laser power of 100mW, aperture of 50 × 1000 µm, a ×100 objective, 3 co-additions (scan repetitions) and an integration time of 35 s. The spectral range was 405–1780 cm^−1^ with a spectral resolution of 3–5 cm^−1^.

All 17 measurements of each sample were averaged to obtain mean values. At the same time, mean values for each measurement position across groups (AML, DIO, BYP) were calculated in order to assess potential differences relative to the measurement position. Subregions were defined as follows:

- *Outer Surface Condyle (Out S.C.)* 5 measurements per sample @ positions 10, 11, 12, 16 and 17, *i.e.* closest to condyle outer surface;
- *Mid Condyle (Mid C.)* 5 measurements per sample @ positions 2, 3, 4, 8 and 9, *i.e.* representing the middle part of the condyle, lying between the condyle outer surface and condyle centre;
- *Centre Condyle (Cen C.)* 7 measurements per sample @ positions 1, 5, 6, 7, 13, 14 and 15, representing the centre of the condyle.

**Fig. 3:**
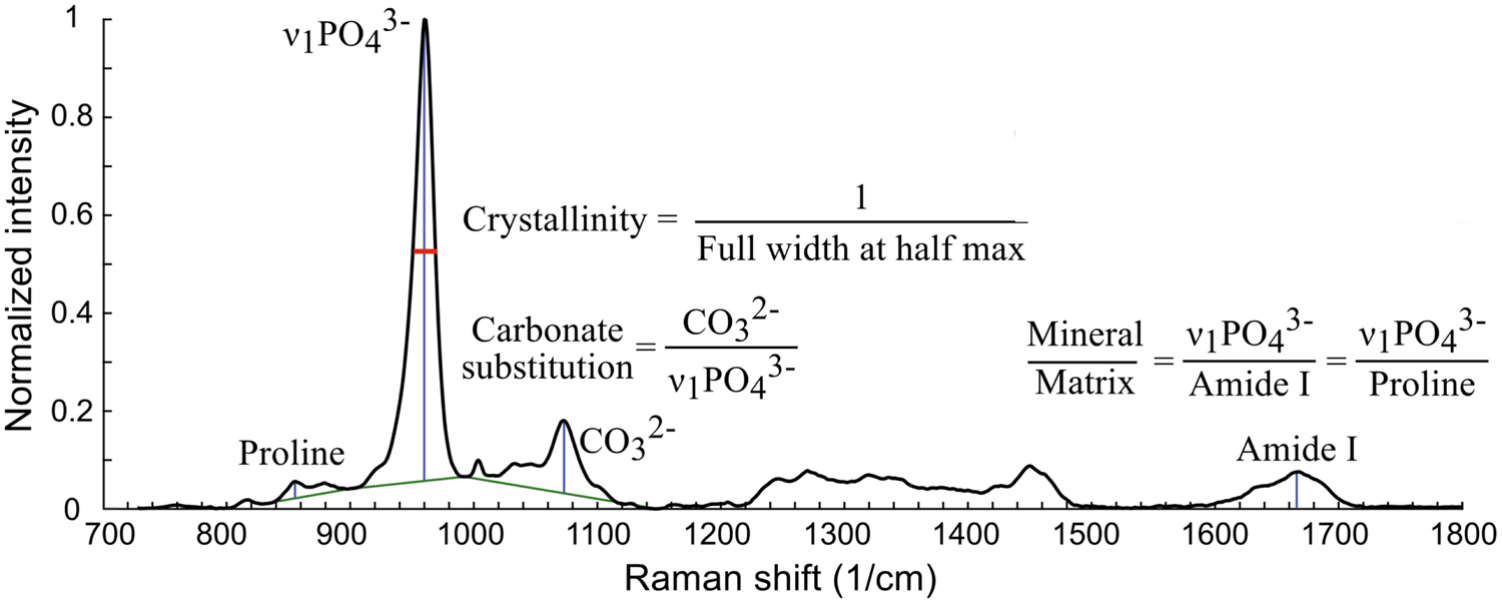
Raman spectrum of bone with calculated peak ratios for the parameters mineralization, carbonate substitution and crystallinity. (From Creecy et al. [7]; with permission)

The spectra were retrieved using OPUS software 7.2 (Bruker, Massachusetts, USA). Following baseline correction, peak intensities (Fig. 3) [7] were calculated for each specific Raman-associated band of interest (displayed in Table 1). These spectra were used to determine the following parameters [3, 32, 33]:

- *Mineralization degree by mineral-to-matrix ratio* obtained by dividing the primary phosphate band (960 cm^−1^) by amide I band (1670 cm^−1^);
- *Carbonate substitution for phosphate position by carbonate-to-phosphate ratio* obtained by dividing the carbonate band (1070 cm^−1^) by the primary phosphate band;
- *Size and perfection of the bone mineral by crystallinity* calculated as the inverse of the full width at half maximum of the primary phosphate band (range between 925 and 990 cm^−1^) using the OriginPro 2017 software (OriginLab, Massachusetts, USA);
- *Organic phase of bone tissue by assessment of the accumulation of AGEs* obtained by normalising pentosidine (1495 cm^−1^) and CML (1150 cm^−1^) band to CH_2_ band (1450 cm^−1^). Additional details regarding the specificity of the Raman spectral areas for CML and pentosidine from a wildtype and a diabetic mouse can be found in Rubin et al. [33].

**Table 1:** Bone mineral and matrix components (left column) with their corresponding Raman spectroscopic band (middle column)

Peak allocation	Peaks (cm^−1^)	Integration interval (cm^−1^)
_v1_PO_4_^3−^	960	918,50–987,15
_v1_CO_3_^2−^	1070	1053,44–1105,52
δ(CH_2_)	1450	1403,80–1491,39
Amide I	1670	1619,22–1709,18
Carboxymethyl-lysine	1150	Individual
Pentosidine	1495	Individual

The right column displays the interval in which the corresponding peak intensity was measured by using OPUS software’s integration function. Carboxymethyl-lysine and pentosidine integration intervals were manually selected for each measurement due to s and interval [3, 32]

### Statistical Analysis

Data for all parameters are expressed as scatter plots with means ± standard deviation. Linear models for repeated measurements were used for data analysis, with the ratio as response variable and either group (aim 1) or locus (aim 2) as explanatory variable. A random intercept was modelled to account for clustering of measurements within animal. In some cases outliers occurred, disturbing the symmetry of the distribution. As a sensitivity analysis, additional analyses were performed with a square root-transformed response variable to improve the symmetry of the distribution. If *p* values from both analyses were similar, it was anticipated that outliers do not influence the results considerably and results based on the raw (untransformed) data can be reported. All tests were two-sided and a 5% significance level was assumed for all analyses. No corrections for multiplicity were performed. Analyses were performed using SAS software (version 9.4 of the SAS system for Windows).

## Results

### Decreased Mineralization is Observed in DIO and BYP Condyles

**Fig. 4:**
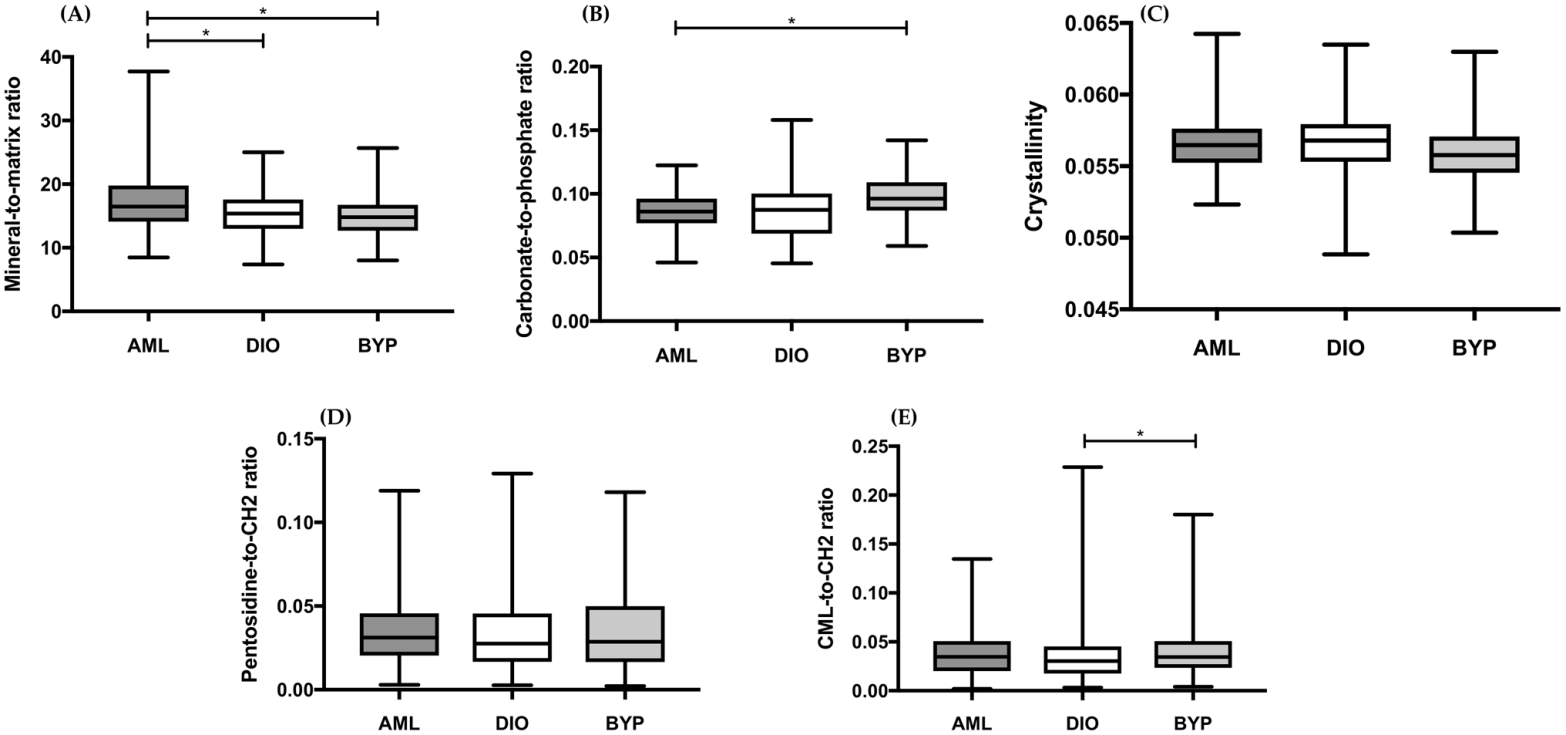
Raman-based mineral-to-matrix ratio (**a**), carbonate-to-phosphate ratio (**b**), crystallinity (**c**), AGE pentosidine (**d**) and AGE CML (**e**) for AML, DIO, and BYP condyle trabecular bone. Boxplots created using GraphPad Prism V8.1.1 *p < 0.05

The trabecular bone mineral-to-matrix ratio was found to be significantly lower for DIO and BYP animals compared to AML animals (Fig. 4a). No significant differences for the degree of mineralization of the condyle could be seen between DIO and BYP samples. Furthermore, the highest carbonate-to-phosphate ratio was found for the BYP condyle, reaching statistical significance when compared to the AML condyle (Fig. 4b). No differences were noted between AML and DIO bone samples for this parameter. Regarding crystallinity, no differences were found between all groups (Fig. 4c). Also, the AGE pentosidine remained unaffected by the experimental treatments (Fig. 4d). In contrast, the other investigated AGE, namely CML, was found significantly increased in the BYP condyle compared to DIO (Fig. 4e).

### The Condyle Bone Composition is Non-uniform and Site-Selection does Matter

**Fig. 5:**
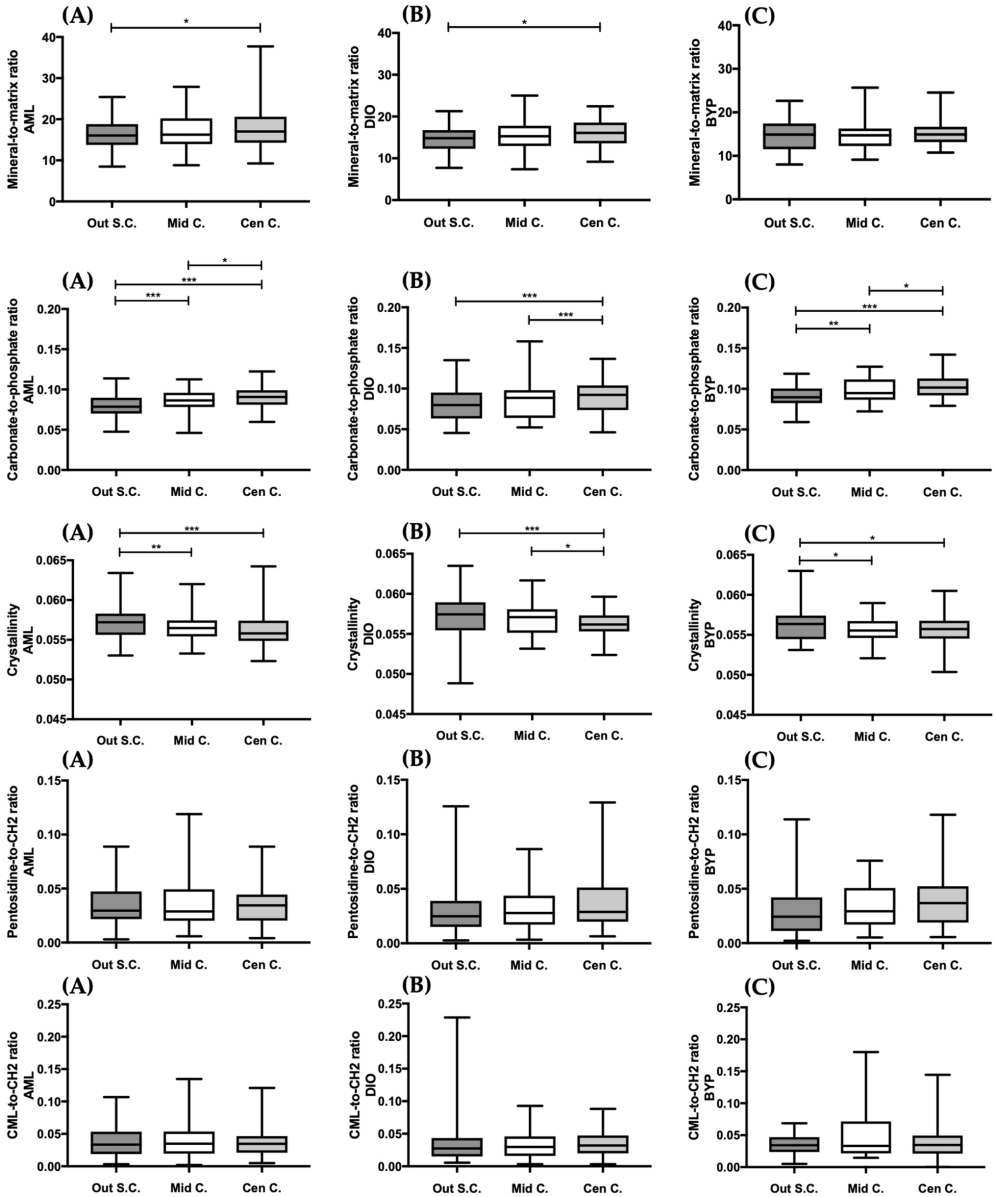
Site differences for Raman-based mineral-to-matrix ratio (first row), carbonate-to-phosphate ratio (second row), crystallinity (third row), pentosidine (fourth row) and CML (fifth row) for AML (**a**), DIO (**b**) and BYP (**c**) groups. *Out S.C.* outer surface condyle, *Mid C* mid condyle, *Cen C* centre condyle. Boxplots created using GraphPad Prism V8.1.1 *p < 0.05; **p < 0.01; ***p < 0.001

DIO and AML mandibular condyle bones presented significant site-specific differences in the degree of mineralization, increasing from the outer surface of the condyle towards the centre (Fig. 5, first row). This was not observed in the BYP group. Significantly more carbonate substitution was observed in the centre of all groups’ condyles, when compared both to the middle and outer surface areas, except for the DIO condyle which did not show a significant difference between the middle and outer surface (Fig. 5, second row). Furthermore, all groups showed a significantly increased crystallinity in the outer surface of the condyle compared to the other measurement positions, except for the DIO group which did not show a significant difference between the middle and outer surface (Fig. 5, third row). Lastly, no significant site-dependency was noted for AGEs accumulation in any of the groups (Fig. 5, fourth and fifth row).

## Discussion

T2DM adversely affects bone in numerous ways and includes a deteriorated bone quality. RYGB surgery is a frequently performed and effective treatment for morbidly obese T2DM patients [43]. However, RYGB-associated side effects also exist with negative impact on the skeleton [47]. Therefore, we aimed at assessing the compositional characteristics of the mandibular condyle bone for these two conditions through Raman spectroscopy.

Raman outcomes reveal bone composition at microscale level, and our results showed an altered mandibular condyle composition in the DIO state. Particularly, mineralization was found to be decreased in T2DM (DIO) samples when compared to controls, perchance instigated by a decreased remodelling rate [25, 35]. But another factor should be considered as a potential cause of the observed change in bone mineralization, namely the food consistency with the HFD pellets being softer than the LFD ones. The hardness of the diet can affect the bone quality of the mandibular condyle, as evidenced by Kufley et al. [22] in Wistar rats. Indeed, soft diet significantly decreased the bone quality of the mandible though only for the subset of animals in periods of active growing. These results are in line with previous investigations displaying a negative effect on the temporomandibular joint in growing animals after altering the function by changing the consistency of the diet [20, 27] or by changing the performance of the masticatory muscles [23]. However, given the fact that the rats used in the present study were aged 30 weeks, we anticipate that the diet hardness was not a confounding variable when interpreting the observed results of decreased mineralization in the T2DM condition. In the same context, we should keep in mind that different animal studies addressing the influence of diet-related altered masticatory function on mandibular condyle are considering different regions of interest, such as the postero-superior area [22], the anterior portion [13], the centre [39] or the full condylar bone (such as in the present study). Different forces are applied to these different areas of the condyle therefore having different outcomes with endochondral ossification and bone deposition occurring more pronounced in the postero-superior area in the mandibular condyle [11].

BYP mice also exhibited a significantly decreased mineralization when compared to control (AML) mice, and moreover displayed an even larger decrease compared to DIO mice, suggesting the T2DM-induced decrease in bone mineralization is not reversed by RYGB treatment. Similar outcomes were obtained for the cortical femoral region lying of diabetic and bypass-treated mice [24]. Carbonate-to-amide I ratio, which has been linked to alterations in bone remodelling favouring bone frailty [14, 28], was increased for BYP compared to DIO bones, which is consistent with our observed decreased mineralization in BYP samples.

Along with mineralization, carbonate substitutions are also known to be a predictor for the mechanical properties of bone [46]. When phosphate is replaced by carbonate, the shape of the mineral crystal is altered with potential detrimental mechanical consequences [1]. In the present study, only carbonate substitutions in BYP bones was significantly increased compared to AML animals, while no differences were detected between AML and DIO animals, suggesting that carbonate substitution is evoked by RYGB treatment. A possible elucidation can be found in the study from Yu et al. [48], where alteration of the gastrointestinal hormones following RYGB surgery with direct effects on the skeleton was observed. Marin et al. [24], in contrast, reported less carbonate substitutions in the femoral trabecular area of obese and bypass compared to lean mice, indicating different responses relative to the bone type. By combining our carbonate substitution and decreased mineralization results within DIO and BYP groups, we can assume that the adverse effect of T2DM on the mechanical properties of the mandibular condyle bone are not resolved by RYGB treatment.

Bone crystallinity shows a relationship with carbonate substitution levels as both are dependent of the primary phosphate band. An increased crystallinity or maturity is mirrored by a decrease in carbonate substitutions, displaying an apatite cell with a greater crystallographic perfection [3]. The latter benefits bone strength and stiffness, but reduces its ability to deform [46], as fully crystallised compounds can be compared to very brittle ceramics [10]. The mandibular condyle crystallinity in the present study remained unaffected by neither the DIO or BYP condition, confirming the carbonate substitution results except from the increase in BYP samples, in which crystallinity was found to be slightly decreased (*p* = 0.45). Further studies with larger sample sizes for confirmation of the latter results are welcomed given the p-value closer to the significance threshold value.

Among the AGEs, pentosidine is widely been used as a surrogate marker [34]. In the present study a trend towards an increased presence of pentosidine in BYP bone compared to both other groups was found. The same was found for CML, which was increased with significance in BYP bone compared to DIO samples. The accumulation of AGEs in bone is interrelated with biomechanical weakness by leading to more brittle bones which are less deformable prior to fracturing [8, 40]. Moreover, as bone matrix proteins are altered through AGEs, they are reported to impede the osteoclastic differentiation process as well, causing the bone resorption process to be decreased with an altered bone homeostasis as consequence [41, 49]. Marin et al. [24] reported for obese and bypass femoral mice bones both an increase in pentosidine and CML compared to AML, which they found to be associated to decreased bone stiffness and strength as revealed by finite elements modelling. These results for long bones, *i.e.* significant differences for pentosidine and CML between AML and both DIO and BYP groups, could not be found for the oral condylar bone in the present study. Therefore and awaiting more studies thereon, the clinical relevance of the AGE results in RYGB treated patients, cautiously suggesting the generation of brittled condyle bones, is very limited. Moreover, oral condyle fractures occur mainly following trauma [5] rather than caused by bone pathology.

Furthermore, to the authors’ knowledge for the first time, compositional variances between different locations within the condyle were assessed. Similar approaches for site-dependency of Raman outcomes were performed before but on the cortical area of femoral bones [15, 28]. Marin et al. [24] revealed that significant decrease of mineralization was limited to the cortical endosteal surface. Our results revealed a decrease from the centre to the outer surface of the condyle throughout all groups, and overall mineralization decline is suggested to originate in the outer layer of the trabecular area, gradually affecting the surrounding trabecular and cortical bone. For carbonate substitution levels, a similar gradual decrease from the centre of the mandibular condyle towards the outer surface is shown and reaches significance for all groups. Therefore, these novel data evidence that the composition of the mandibular condyle is not uniform, with significant site differences for all experimental groups, anticipating that the youngest bone can be found at the outer surface of the trabecular condyle, characterised by lower mineralization and carbonate substitution levels [1].

## Conclusion

The current study on the mandibular condyle bone using Raman spectroscopy reveals novel data on alterations in the oral condyle bone composition of T2DM (DIO) and RYGB treated (BYP) mice. The mineralization degree in both diabetic and bariatric mandibular condyle was found significantly lower compared to age-matched controls, weakening the bone. In addition, the BYP group showed elevated carbonate substitution levels compared to controls. Accumulation of the AGE CML, known to be related to the bone toughness [17, 34], occurred in the mandibular condyle of BYP mice, though with unclear interpretation. To further evidence our results, histological and microfocus X-ray-computed tomography studies of the DIO and BYP mandibular condyle bone structure would be of interest and is currently lacking in the field. Site-specific measurements revealed a non-uniform condyle bone composition within all groups, with highest mineralization centrally and a coupled decrease and increase of carbonate substitution and crystallinity respectively at the outer surface. Further experimentation on bone remodelling, structural characteristics and mechanical tests such as nanoindentation of the mandibular condyle in T2DM and bariatric conditions should be conducted to confirm the effects of these pathologies on this area of the skeleton.

## Electronic supplementary material

Below is the link to the electronic supplementary material.

Supplementary file1 (DOCX 39 kb)

## Data Availability

See online supplementary tables for original statistical analysis data, or contact for original measurement data.

## References

[1] Akkus O, Adar F, Schaffler MB (2004). Age-related changes in physicochemical properties of mineral crystals are related to impaired mechanical function of cortical bone. Bone.

[2] Ammann P, Rizzoli R (2003). Bone strength and its determinants. Osteoporos Int.

[3] Awonusi A, Morris MD, Tecklenburg MMJ (2007). Carbonate assignment and calibration in the raman spectrum of apatite. Calcif Tissue Int.

[4] Bazin D, Chappard C, Combes C, Carpentier X, Rouzière S, André G, Matzen G (2009). Diffraction techniques and vibrational spectroscopy opportunities to characterise bones. Osteoporos Int.

[5] Chandra L, Deepa D, Atri M, Pandey SM, Passi D, Goyal J, Sharma A, Gupta U (2019). A retrospective cross-sectional study of maxillofacial trauma in Delhi-NCR Region. J Family Med Prim Care.

[6] Cho NG, Shaw JE, Karuranga S, Huang Y, da Rocha Fernandes JD, Ohlrogge AW, Malanda B (2018). IDF Diabetes atlas: global estimates of diabetes prevalence for 2017 and projections for 2045. Diabetes Res Clin Pract.

[7] Creecy A, Uppuganti S, Merkel AR, O'Neal D, Makowski AJ, Granke M, Voziyan P (2016). Changes in the fracture resistance of bone with the progression of type 2 diabetes in the ZDSD rat. Calcif Tissue Int.

[8] Devlin MJ, Van Vliet M, Motyl K, Karim L, Brooks DJ, Louis L, Conlon C (2014). Early-onset type 2 diabetes impairs skeletal acquisition in the male TALLYHO/JngJ mouse. Endocrinology.

[9] Freeman JJ, Silva MJ (2003). Separation of the Raman spectral signatures of bioapatite and collagen in compact mouse bone bleached with hydrogen peroxide. Appl Spectrosc.

[10] Fu X, Chen J, Wu D, Du Z, Lei Q, Cai Z, Schultze-Mosgau S (2012). Effects of ovariectomy on rat mandibular cortical bone: a study using raman spectroscopy and multivariate analysis. Anal Chem.

[11] Fuentes MA, Opperman LA, Buschang P, Bellinger LL, Carlson DS, Hinton RJ (2003). Lateral functional shift of the mandible: Part I. Effects on condylar cartilage thickness and proliferation. Am J Orthod Dentofacial Orthop..

[12] Goodyear SR, Gibson IR, Skakle JMS, Wells RPK, Aspden RM (2009). A comparison of cortical and trabecular bone from C57 Black 6 mice using Raman spectroscopy. Bone.

[13] Grünheid T, Langenbach GE, Brugman P, Everts V, Zentner A (2011). The masticatory system under varying functional load. Part 2: effect of reduced masticatory load on the degree and distribution of mineralization in the rabbit mandible. Eur J Orthod..

[14] Inzana JA, Maher JR, Takahata M, Schwarz EM, Berger AJ, Awad HA (2013). Bone fragility beyond strength and mineral density: raman spectroscopy predicts femoral fracture toughness in a murine model of rheumatoid arthritis. J Biomech.

[15] Jordan GR, Loveridge N, Bell KL, Power J, Rushton N, Reeve J (2000). Spatial clustering of remodeling osteons in the femoral neck cortex: a cause of weakness in hip fracture?. Bone.

[16] Judex S, Boyd S, Qin YX, Miller L, Müller R, Rubin C (2003). Combining high-resolution micro-computed tomography with material composition to define the quality of bone tissue. Curr Osteoporos Rep.

[17] Karim L, Bouxsein ML (2016). Effect of type 2 diabetes-related non-enzymatic glycation on bone biomechanical properties. Bone.

[18] Kaul R, O’Brien MH, Dutra E, Lima A, Utreja A, Yadav S (2016). The effect of altered loading on mandibular condylar cartilage. PLoS ONE.

[19] Khan AF, Awais M, Khan AS, Tabassum S, Chaudhry AA, Rehman IU (2013). Raman spectroscopy of natural bone and synthetic apatites. Appl Spectrosc Rev.

[20] Kiliaridis S (1995). Masticatory muscle influence on craniofacial growth. Acta Odontol Scand.

[21] Kim JH, Lee DE, Gunawardhana KS, Choi SH, Woo GH, Cha JH, Bak EJ (2014). Effect of the interaction between periodontitis and type 1 diabetes mellitus on alveolar bone, mandibular condyle and tibia. Acta Odontol Scand.

[22] Kufley S, Scott JE, Ramirez-Yanez G (2017). The effect of the physical consistency of the diet on the bone quality of the mandibular condyle in rats. Arch Oral Biol.

[23] Kün-Darbois JD, Libouban H, Chappard D (2015). Botulinum toxin in masticatory muscles of the adult rat induces bone loss at the condyle and alveolar regions of the mandible associated with a bone proliferation at a muscle enthesis. Bone.

[24] Marin C, Papantonakis G, Sels K, van Lenthe GH, Falgayrac G, Vangoitsenhoven R, Van Der Schueren B (2018). Unraveling the compromised biomechanical performance of type 2 diabetes- and Roux-en-Y gastric bypass bone by linking mechanical-structural and physico-chemical properties. Sci Rep.

[25] Marin C, Luyten FP, Van der Schueren B, Kerckhofs G, Vandamme K (2018). The impact of type 2 diabetes on bone fracture healing. Front Endocrinol.

[26] Marugame T, Hayasaki H, Lee K, Eguchi H, Matsumoto S (2003). Alveolar bone loss associated with glucose tolerance in Japanese men. Diabet Med.

[27] Mavropoulos A, Ammann P, Bresin A, Kiliaridis S (2005). Masticatory demands induce region-specific changes in mandibular bone density in growing rats. Angle Orthod.

[28] McCreadie BR, Morris MD, Chen TC, Sudhaker Rao D, Finney WF, Widjaja E, Goldstein SA (2006). Bone tissue compositional differences in women with and without osteoporotic fracture. Bone.

[29] Morris MD, Mandair GS (2011). Raman assessment of bone quality. Clin Orthop Relat Res.

[30] Mosekilde L (1998). The effect of modelling and remodelling on human vertebral body architecture. Technol Health Care.

[31] Onoyama K, Kawamata R, Kozai Y, Sakurai T, Kashima I (2011). Comparison of mandibular trabecular structures between normal and diabetic rats: evaluation of spontaneous type 2 diabetes in a rat model. Oral Radiol.

[32] Penel G, Delfosse C, Descamps M, Leroy G (2005). Composition of bone and apatitic biomaterials as revealed by intravital Raman microspectroscopy. Bone.

[33] Rubin MR, Paschalis EP, Poudarik A, Sroga GE, McMahon DJ, Gamsjaeger S, Klaushofer K (2016). Advanced glycation endproducts and bone material properties in type 1 diabetic mice. PLoS ONE.

[34] Saito M, Marumo K (2010). Collagen cross-links as a determinant of bone quality: a possible explanation for bone fragility in aging, osteoporosis, and diabetes mellitus. Osteoporos Int.

[35] Sanches CP, Vianna AGD, Barreto FC (2017). The impact of type 2 diabetes on bone metabolism. Diabetol Metab Syndr.

[36] Schernthaner G, Brix JM, Kopp HP, Schernthaner GH (2011). Cure of type 2 diabetes by metabolic surgery? A critical analysis of the evidence in 2010. Diabetes Care.

[37] Shanbhogue VV, Mitchell DM, Rosen CJ, Bouxsein ML (2016). Type 2 diabetes and the skeleton: new insights into sweet bones. Lancet Diabetes Endocrinol.

[38] Tamayo T, Rosenbauer J, Wild SH, Spijkerman AM, Baan C, Forouhi NG, Herder C (2014). Diabetes in Europe: An update. Diabetes Res Clin Pract.

[39] Tanaka E, Sano R, Kawai N, Langenbach GE, Brugman P, Tanne K, van Eijden TM (2007). Effect of food consistency on the degree of mineralization in the rat mandible. Ann Biomed Eng.

[40] Tang SY, Allen MR, Phipps R, Burr DB, Vashishth D (2009). Changes in non-enzymatic glycation and its association with altered mechanical properties following 1-year treatment with risedronate or alendronate. Osteoporos Int.

[41] Valcourt U, Merle B, Gineyts E, Viguet-Carrin S, Delmas PD, Garnero P (2007). Non-enzymatic glycation of bone collagen modifies osteoclastic activity and differentiation. J Biol Chem.

[42] Vangoitsenhoven R (2016) The role of diet in development and reversal of type 2 diabetes [dissertation]. [Leuven (Belgium)]: KU Leuven.

[43] Vasas P, Al-khyatt W, Idris I, Leeder PC, Awan AK, Awad S, Ahmed J (2016). Mid-term remission of type 2 diabetes mellitus after laparoscopic Roux En-Y gastric bypass. World J Surg.

[44] Vestergaard P (2007). Discrepancies in bone mineral density and fracture risk in patients with type 1 and type 2 diabetes—a meta-analysis. Osteoporos Int.

[45] Yamagishi S, Maeda S, Matsui T, Ueda S, Fukami K, Okuda S (2012). Role of advanced glycation end products (AGEs) and oxidative stress in vascular complications in diabetes. Biochim Biophys Acta.

[46] Yerramshetty JS, Akkus O (2008). The associations between mineral crystallinity and the mechanical properties of human cortical bone. Bone.

[47] Yu EW (2014). Bone metabolism after bariatric surgery. J Bone Miner Res.

[48] Yu EW, Wewalka M, Ding SA, Simonson DC, Foster K, Holst JJ, Vernon A (2016). Effects of gastric bypass and gastric banding on bone remodeling in obese patients with type 2 diabetes. J Clin Endocrinol Metab.

[49] Zhang L, Chen B, Tang L (2012). Metabolic memory: mechanisms and implications for diabetic retinopathy. Diabetes Res Clin Pract.

