# Supplementary material for: Alteration of the Condylar Oral Bone in Obese and Gastric Bypass Mice
Source: Calcif Tissue Int. 2020 Aug 1;107(4):371–80. doi: 10.1007/s00223-020-00732-0 (PMC7497324; doi:10.1007/s00223-020-00732-0)
Supplement: Supplementary file 1 — Supplementary file1 (DOCX 39 kb) [file 223_2020_732_MOESM1_ESM.docx]

**Alteration of the condylar oral bone in obese and gastric bypass mice**

**[Supplementary Tables]**

*Nicolas Colsoul^1^*, Carlos Marin^1,2,3^*, Katrien Corbeels^4^, Greet Kerckhofs^2,5,6,7^, Bart Van der Schueren^4^, Katleen Vandamme^1,2#^. (*Shared first authorship)*

^1^ Biomaterials – BIOMAT, Department of Oral Health Sciences, KU Leuven, Leuven, Belgium.

^2^ Prometheus – Division of Skeletal Tissue Engineering Leuven, KU Leuven, Leuven, Belgium.

^3^ Skeletal Biology and Engineering Research Center, Department of Development and Regeneration, KU Leuven, Leuven, Belgium.

^4^ Clinical and Experimental Endocrinology, Department of Chronic Diseases, Metabolism and Ageing (CHROMETA),, KU Leuven, Leuven, Belgium.

^5^ Department of Material Science and Engineering, KU Leuven, Leuven, Belgium.

^6^ Biomechanics lab, Institute of Mechanics, Materials, and Civil Engineering, UCLouvain, Louvain-la-Neuve, Belgium.

^7^ Institute for Experimental and Clinical Research, UClouvain, Woluwe, Belgium.

^8^ Leuven Biostatistics and Statistical Bioinformatics Center (L-Biostat) , KU Leuven, Leuven, Belgium.

^#^Corresponding author: Prof. Katleen Vandamme; Biomaterials; BIOMAT, Department of Oral Health Sciences, KU Leuven, Leuven, Belgium; Tel: (+32)16332438; Email: [katleen.vandamme@uzleuven.be](mailto:katleen.vandamme@uzleuven.be)

**Supplementary Table 1** Statistical analysis of Raman-based differences in mineral-to-matrix ratio, carbonate-to-phosphate ratio, crystallinity, AGE pentosidine and AGE CML between AML and DIO (upper), AML and BYP (middle), BYP and DIO (lower) groups.

|  | **Mean (95% CI)** | |  | | |
| --- | --- | --- | --- | --- | --- |
| **Ratio** | **Control** | **Diabetes** | **Difference** | **P-value** | **P-value*** |
| Carbonate to Phosphate | 0.086 (0.078;0.094) | 0.085 (0.077;0.093) | -0.001 (-0.012;0.011) | 0.9016 | 0.7954 |
| Phosphate to Amide I | 17.403 (16.051;18.754) | 15.347 (14.050;16.643) | -2.056 (-3.928;-0.183) | 0.0315 | 0.0379 |
| Pentosidine to CH2 | 0.032 (0.027;0.037) | 0.032 (0.027;0.037) | -0.001 (-0.008;0.007) | 0.8860 | 0.7585 |
| Crystallinity | 0.057 (0.056;0.058) | 0.057 (0.056;0.058) | 0.000 (-0.001;0.001) | 0.7882 | 0.7827 |
| CML to CH2 | 0.036 (0.032;0.041) | 0.032 (0.028;0.037) | -0.004 (-0.010;0.002) | 0.1947 | 0.1147 |

| **Ratio** | **BYP** | **DIO** | **Difference** | **P-value** | **P-value*** |
| --- | --- | --- | --- | --- | --- |
| Carbonate to Phosphate | 0.097 (0.086;0.108) | 0.085 (0.077;0.093) | -0.012 (-0.026;0.002) | 0.0820 | 0.0734 |
| Phosphate to Amide I | 14.942 (13.869;16.014) | 15.353 (14.531;16.175) | 0.411 (-0.940;1.762) | 0.5498 | 0.5726 |
| Pentosidine to CH2 | 0.036 (0.028;0.043) | 0.032 (0.026;0.038) | -0.004 (-0.014;0.006) | 0.4185 | 0.3166 |
| Crystallinity | 0.056 (0.055;0.057) | 0.057 (0.056;0.058) | 0.001 (-0.001;0.002) | 0.2505 | 0.2483 |
| CML to CH2 | 0.041 (0.034;0.048) | 0.032 (0.027;0.038) | -0.009 (-0.017;-0.000) | 0.0435 | 0.0116 |

| **Ratio** | **AML** | **BYP** | **Difference** | **P-value** | **P-value*** |
| --- | --- | --- | --- | --- | --- |
| Carbonate to Phosphate | 0.086 (0.080;0.091) | 0.097 (0.090;0.104) | 0.012 (0.003;0.021) | 0.0122 | 0.0125 |
| Phosphate to Amide I | 17.403 (16.011;18.794) | 14.941 (13.196;16.685) | -2.462 (-4.693;-0.231) | 0.0307 | 0.0345 |
| Pentosidine to CH2 | 0.032 (0.027;0.037) | 0.036 (0.029;0.042) | 0.003 (-0.005;0.011) | 0.4052 | 0.4193 |
| Crystallinity | 0.057 (0.056;0.058) | 0.056 (0.055;0.057) | -0.001 (-0.002;0.001) | 0.4547 | 0.4529 |
| CML to CH2 | 0.036 (0.031;0.041) | 0.041 (0.035;0.047) | 0.005 (-0.003;0.013) | 0.2419 | 0.1813 |
| CI: confidence interval. *P-value for transformed outcome | | | | | |

|  |
| --- |

**Supplementary Table 2** Statistical analysis of Raman-based differences in mineral-to-matrix ratio, carbonate-to-phosphate ratio, crystallinity, AGE pentosidine and AGE CML for the different regions [Out S.C.: Outer surface condyle, Mid C.: mid condyle, Cen C.: centre condyle] for control (upper), diabetes (middle) and bypass (lower) groups.

|  | **Mean (95% CI)** | | |  | **Pairwise P-values** | | |  |
| --- | --- | --- | --- | --- | --- | --- | --- | --- |
| **Ratio** | **Out S.C. AML** | **Mid C. AML** | **Cen C. AML** | **Global P-value** | **1-2** | **1-3** | **2-3** | **Global P-value*** |
| Carbonate to Phosphate | 0.080 (0.074;0.086) | 0.086 (0.080;0.092) | 0.090 (0.084;0.095) | <.0001 | 0.0008 | <.0001 | 0.0289 | <.0001 |
| Phosphate to Amide I | 16.417 (14.514;18.319) | 17.366 (15.469;19.263) | 18.118 (16.299;19.937) | 0.0435 | 0.1933 | 0.0125 | 0.2633 | 0.0513 |
| Pentosidine to CH2 | 0.033 (0.027;0.039) | 0.035 (0.029;0.041) | 0.030 (0.024;0.035) | 0.3297 | 0.5380 | 0.4178 | 0.1410 | 0.2953 |
| Crystallinity | 0.057 (0.056;0.058) | 0.056 (0.055;0.058) | 0.056 (0.055;0.057) | 0.0004 | 0.0028 | 0.0001 | 0.5130 | 0.0003 |
| CML to CH2 | 0.036 (0.030;0.043) | 0.036 (0.030;0.043) | 0.036 (0.030;0.042) | 0.9981 | 0.9999 | 0.9590 | 0.9592 | 0.9886 |
| **Ratio** | **Out S.C. DIO** | **Mid C. DIO** | **Cen C. DIO** | **Global P-value** | **1-2** | **1-3** | **2-3** | **Global P-value*** |
| Carbonate to Phosphate | 0.079 (0.069;0.089) | 0.083 (0.073;0.093) | 0.091 (0.081;0.100) | <.0001 | 0.0680 | <.0001 | 0.0009 | <.0001 |
| Phosphate to Amide I | 14.602 (13.460;15.744) | 15.226 (14.095;16.357) | 15.958 (14.908;17.009) | 0.0384 | 0.2775 | 0.0116 | 0.1661 | 0.0337 |
| Pentosidine to CH2 | 0.031 (0.023;0.039) | 0.030 (0.022;0.038) | 0.033 (0.026;0.041) | 0.6854 | 0.9066 | 0.5116 | 0.4294 | 0.8835 |
| Crystallinity | 0.057 (0.057;0.058) | 0.057 (0.056;0.058) | 0.056 (0.055;0.057) | 0.0001 | 0.1036 | <.0001 | 0.0108 | 0.0001 |
| CML to CH2 | 0.032 (0.025;0.040) | 0.030 (0.023;0.038) | 0.034 (0.027;0.040) | 0.7614 | 0.6915 | 0.7611 | 0.4609 | 0.5414 |
| **Ratio** | **Out S.C. BYP** | **Mid C. BYP** | **Cen C. BYP** | **Global P-value** | **1-2** | **1-3** | **2-3** | **Global P-value*** |
| Carbonate to Phosphate | 0.090 (0.082;0.098) | 0.097 (0.089;0.106) | 0.102 (0.094;0.111) | <.0001 | 0.0034 | <.0001 | 0.0322 | <.0001 |
| Phosphate to Amide I | 14.636 (13.417;15.856) | 14.932 (13.699;16.166) | 15.170 (14.094;16.245) | 0.7545 | 0.7021 | 0.4541 | 0.7412 | 0.6976 |
| Pentosidine to CH2 | 0.033 (0.022;0.043) | 0.033 (0.023;0.044) | 0.039 (0.030;0.049) | 0.3682 | 0.9573 | 0.2225 | 0.2493 | 0.3315 |
| Crystallinity | 0.057 (0.055;0.058) | 0.056 (0.055;0.057) | 0.056 (0.055;0.057) | 0.0187 | 0.0172 | 0.0104 | 0.9778 | 0.0206 |
| CML to CH2 | 0.035 (0.023;0.047) | 0.047 (0.035;0.059) | 0.041 (0.031;0.052) | 0.2374 | 0.0912 | 0.3212 | 0.3968 | 0.7163 |
| CI: confidence interval. *Global P-value for transformed outcome. Pairwise tests: 1=Out S.C., 2=Mid C., 3=Cen C. | | | | | | | | |
